# Laboratory tests for the detection of SARS-CoV-2 infection: basic principles and examples

**DOI:** 10.3205/000293

**Published:** 2021-05-27

**Authors:** Khaled R. Alkharsah

**Affiliations:** 1 Department of Microbiology, College of Medicine, Imam Abdulrahman Bin Faisal University (IAU), Dammam, Saudi Arabia

**Keywords:** COVID-19, SARS-CoV-2, coronavirus, laboratory test, molecular assay, immunoassay

## Abstract

Severe acute respiratory syndrome coronavirus 2 (SARS-CoV-2) has circulated throughout the world causing the worst pandemic since 1918. All efforts have been marshalled towards testing different treatment approaches, obtaining clinical and epidemiological information, developing suitable diagnostic tests, and developing new vaccines. New ribonucleic acid (RNA)-based and viral vector-based vaccines have been developed and licensed under emergency use in many countries; however, there is a huge demand for vaccines, and it will take some time before a sufficient number of people are vaccinated to stop the circulation of the virus. Therefore, the proper diagnosis and identification of infected individuals are crucial for the isolation and treatment of these patients and tracing of their contacts. Many diagnostic tests and diag-nostic kits have been developed in a relatively short time. This review summarizes the principles of the available laboratory assays that are in use for the detection of SARS-CoV-2 RNA, antigens, or antibodies.

## Zusammenfassung

Das Severe Acute Respiratory Syndrome Coronavirus 2 (SARS-CoV-2), das sich weltweit ausgebreitet hat, ist für eine der größten Pandemien seit 1918 verantwortlich. Seit Beginn der Pandemie laufen weltweit zahlreiche klinische und epidemiologische Studien, um mögliche effektive Behandlungsansätze zu testen und geeignete diagnostische Testverfahren zu entwickeln. Obwohl in einigen Ländern bereits einige RNA- und Vektorimpfstoffe gegen das Virus entwickelt und zugelassen wurden, ist die Nachfrage nach Impfstoffen in vielen Ländern immer noch groß. Aus diesem Grund und bis eine ausreichende Zahl von Menschen weltweit geimpft ist, bleibt eine schnelle Diagnose die entscheidende Maßnahme, um infizierte Personen zu identifizieren und das Infektionsgeschehen zurückzuverfolgen. In relativ kurzer Zeit wurden viele diagnostische Testverfahren und diagnostische Kits entwickelt, die den Nachweis von SARS-CoV-2-RNA, -Antigenen oder -Antikörpern ermöglichen. Dieser Review-Artikel gibt eine Übersicht über verfügbare diagnostische COVID-19-Tests, ihre Prinzipien und Einsatzmöglichkeiten.

## 1 Introduction

The pandemic of coronavirus disease 2019 (COVID-19) continues to evolve during 2020 affecting life, health, and economy worldwide, making it the worst pandemic since the influenza pandemic in 1918. In addition to millions of infections and millions of deaths, many millions of people have lost their jobs [1]. Severe acute respiratory syndrome coronavirus 2 (SARS-CoV-2), the causative agent of COVID-19, emerged in China at the end of 2019 causing a cluster of pneumonia cases and later spread worldwide [2], [3]. SARS-CoV-2 shares genetic homology with the bat coronaviruses from the genus betacoronavirus, family Coronaviridae, and the previously emerged human coronaviruses; severe acute respiratory syndrome Coronavirus (SARS-CoV) and the Middle East respiratory syndrome Coronavirus (MERS-CoV) [2], [3]. The new coronavirus was found to share 79% genetic similarity with the SARS-CoV and only 50% genetic homology with the MERS-CoV, hence its name of SARS-CoV-2 [4].

The early diagnosis of infected individuals and tracing of their contacts, to isolate them and protect susceptible individuals, are integral factors to controlling infection and containing the pandemic. COVID-19 signs and symptoms such as fever, dry cough, fatigue, and shortness of breath are common among other respiratory tract infections such as SARS, MERS, and influenza [5]. Moreover, patients with COVID-19 presented with diverse clinical symptoms based on the severity of their case, such as sore throat, diarrhea, vomiting, and abdominal pain [5]. Therefore, a clinical diagnosis despite being suggestive is not conclusive of COVID-19. Presymptomatic and asymptomatic transmission of SARS-CoV-2 are missed by clinical diagnosis and contribute to the aggravated spread of the virus and increasing the morbidity and mortality of the disease [6]. The surveillance program, therefore, is a priority to rapidly identify cases and allow contact tracing, isolate the infected individuals, and identify the regional spread of the virus.

Laboratory diagnostic tests can identify apparent and asymptomatic infections and facilitate surveillance programs by detecting the viral nucleic acid, the viral antigens, or the antibodies against the viral antigens. The laboratory diagnosis of SARS-CoV-2 infection can not only play a role in identifying new infections but also in understanding the spread of the disease, the level of its containment in a community, and the level of immunity in a population.

Since the emergence of SARS-CoV-2, unprecedented efforts have been made to develop different types of laboratory assays at exceptional speeds. A laboratory developed test (LDT) for the detection of the viral RNA was available very soon after the announcement of the viral genome sequence [7]. Later, multiple molecular and immunoassays were developed and approved for diagnostic detection of SARS-CoV-2 infection. This review describes the principle of laboratory tests currently available for the detection of COVID-19 infection and summarizes their utility and spectrum of use.

## 2 General laboratory tests and their interpretation

Several blood parameters have been used to reflect the association with SARS-CoV-2 infection. In general, patients infected with SARS-CoV-2 have normal or more commonly decreased leukocyte counts with lymphocytopenia [8], [9], [10], [11], [12]. However, this was not found to be consistent throughout the course of infection. The leukocyte total and differential count vary between mild and severe infections. With progression to severe COVID-19 symptoms, patients tend to have increased leukocyte counts with lymphocytopenia [13], [14], [15]. Lymphocytopenia seems to be a hallmark of COVID-19 severity. Tan et al. suggested a model whereby the percentage of lymphocytes can be used as a predictor of disease prognosis [16]. A lymphocyte percentage higher than 20% would be associated with recovery while a lymphocyte percentage lower than 5% after 17 days of symptoms indicates that the patient is critically ill and needs ICU admission with a high possibility of death [16]. The mechanism by which lymphocytopenia occurs during COVID-19 has not yet been fully studied. It may be due to the direct infection and destruction of lymphocytes by the virus, resulting from their expression of the angiotensin-converting enzyme 2 (ACE2) receptor [17]. Lymphocytes may also be killed by apoptosis induced by several proinflammatory cytokines produced during the course of the disease such as tumor necrosis factor-alpha [18], [19]. Leukocytosis is also accompanied by increased neutrophil counts in severe COVID-19 cases compared to mild cases [15].

C-reactive protein (CRP) is an acute-phase protein expressed at high concentrations during acute inflammatory infection [20]. CRP has been reported to be high in all patients during the course of COVID-19 [10], [21]. However, the CRP levels were lower in children than in adult patients, which may correlate with the milder symptoms observed in children [22]. CRP levels proportionally correlate with the disease severity and diameter of the lung lesions, being highest in critically ill patients with large lung lesions and lower in milder groups with smaller lung lesions [23]. Serum amyloid A (SAA) is another acute-phase protein produced by the liver. It has also been found to increase in severe COVID-19 cases and predicts a poor prognosis [24], [25]. Increased CRP and SAA expression during acute inflammation is usually driven by inflammatory cytokines such as interleukin 6 (IL-6). IL-6 has repeatedly been reported to be high in critically ill COVID-19 patients. Therefore, several studies have suggested using IL-6 as a prognostic biomarker for the development of severe symptoms [26], [27], [28]. Moreover, IL-6 levels were found to be higher in deteriorating patient cases than in patients discharged after SARS-CoV-2 infection [29]. Therefore, it has been suggested to use an IL-6 inhibitor for the treatment of severe COVID-19 cases [30]. Tocilizumab, an anti-IL-6 receptor antibody, was found to produce a rapid and sustained improvement in patients with COVID-19 pneumonia and hyperinflammatory syndrome [31].

D-dimer is a product of fibrin breakdown during the degradation of vascular thrombi and is therefore a good indicator of hemostatic abnormalities and intravascular thrombosis [32]. Normal levels of D-dimer can rule out a suspected pulmonary embolism or deep vein thrombosis in patients with low risk [33]. High levels of D-dimer are indicative of venous thromboembolism but also of other conditions such as pregnancy, impaired renal function, and atrial fibrillation [33]. In severe COVID-19 cases, the risk of developing pulmonary embolism increases with disease progression after the first indication of high D-dimer levels [34]. D-dimer levels were also found to stay high or increase in the non-survivor group of COVID-19 patients compared to survivors [35]. D-dimer levels higher than a cutoff of 1,570 ng/mL were suggestive of asymptomatic deep vein thrombosis in patients with COVID-19 pneumonia [36]. Moreover, COVID-19 patients with D-dimer levels higher than 2,000 ng/mL on hospital admission had a higher incidence of death compared to patients with lower D-dimer levels [37]. 

Other blood biomarkers have also been found to be elevated in COVID-19 patients; however, these other blood biomarkers are not specific indicators of infection but are more useful in the prognosis of disease progression.

## 3 Molecular testing

Suitable samples and proper sampling are crucial for the molecular detection of respiratory viruses. Different types of respiratory tract samples are used to diagnose SARS-CoV-2 infection. A higher rate of positive results is obtained from lower respiratory tract samples; therefore, sputum samples are the better samples when available [38]. If a sputum sample is not available, other upper respiratory tract samples such as nasopharyngeal swabs, oropharyngeal swabs, throat swabs, nasal swabs, or saliva can be used for the detection of SARS-CoV-2 RNA. Nasopharyngeal swabs seem to have a better yield than oropharyngeal and throat swabs detecting more than 80% of cases [38], [39]. Saliva samples are considered a non-invasive alternative for nasopharyngeal swabs, as the two samples show more than 97% agreement [40]. Thermal inactivation of samples prior to testing as a form of personal protection is reported to reduce the RNA copy number by approximately 50% and TRIzol inactivation by about 40%; therefore, it is not recommended [41], [42]. The commonly used nucleic acid extraction protocols and the available nucleic acid extraction kits include a lysis step which is expected to inactivate viruses. However, the efficiency of inactivation varies depending on the lysis buffers employed and the type of sample; therefore, the samples should be considered as potentially infectious [43].

### 3.1 Real-time reverse transcriptase polymerase chain reaction (rRT-PCR)

**Figure 1:**
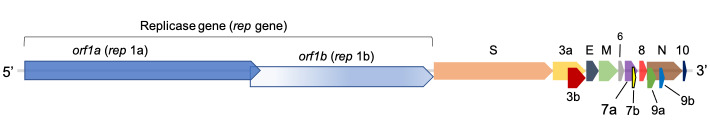
Schematic representation of SARS-CoV-2 genome organization (based on [3])

The real-time reverse transcriptase polymerase chain reaction (rRT-PCR) is, thus far, the gold standard test for the detection of SARS-CoV-2 RNA in clinical samples and plays an important role in the identification of cases [44]. Several protocols have been designed to target different fragments of the viral genome including genes encoding structural proteins, such as spike (S), transmembrane (M), envelope (E), and nucleocapsid (N), or species-specific genes encoding functional proteins such as the RNA-dependent RNA polymerase (RdRp), open reading frame 1a (ORF1a), and ORF1b (Figure 1 (Fig. 1)). The World Health Organization (WHO) recommends the laboratory confirmation of the COVID-19 case by nucleic acid amplification test (NAAT)-based assays in two different spatial categories. In areas with no known circulation of SARS-CoV-2, positive assay results from two different genomic targets specific for SARS-CoV-2 are required to confirm the COVID-19 case, or one positive result for a genomic target from the *Betacoronavirus *genus and sequencing of another genomic fragment for SARS-CoV-2 [45]. For areas with a wide spread of SARS-CoV-2, a positive result from one SARS-CoV-2 discriminatory genomic target is considered sufficient [45]. The American Food and Drug Administration (FDA) has approved the use of over 65 in vitro diagnostic kits for the detection of SARS-CoV-2 under Emergency Use Authorizations (EUA) (FDA-approved kits). These kits are authorized for diagnostic use during the existing circumstances and require further validation. Table 1 (Tab. 1) summarizes a selection of these kits.

While a positive rRT-PCR result for SARS-CoV-2 with clinical manifestation of the disease confirms a COVID-19 case, a negative rRT-PCR does not necessarily preclude infection in the presence of suggestive symptoms. Early studies from China showed that rRT-PCR detected a lower number of COVID-19 cases than chest computerized tomography (CT) [46], [47]. A similar experience was observed with MERS [48]. PCR sensitivity is reported to fade during the course of the infection, being more than 80% during the first 3 days of infection and reaching 45% after day 15; therefore, multiple testing is recommended [49], [50], [51]. The reasons for low PCR sensitivity could be either preanalytical or analytical. Preanalytical causes of low sensitivity range from the type of sample and improper sampling procedures to bad storage and transport [52]. Analytical reasons are mainly attributed to variation between diagnostic assays, which may have been inadequately validated due to the emergency release and approval of the assays [52].

Several studies have compared commercially available assays. A comparison between multiple commercially available kits (from Cepheid, DiaSorin, Hologic Panther, and Roche Cobas) and the protocol designed by the Centers for Disease Control and Prevention (CDC) for SARS-CoV-2 detection based on the N1 and N2 regions showed that all of the assays were 100% specific, and the Cepheid kit had 100% agreement with the CDC protocol [53]. The Abbott Real-Time SARS-CoV-2 assay was found to detect more positive cases than the modified CDC 2019-nCoV RT-PCR [54]. The Cobas SARS-CoV-2 Test detected 4.2% more positive cases than a laboratory-developed assay based on the CDC protocol targeting the N1 and N2 regions of the viral genome [55]. The Cobas 6800 SARS-CoV-2 Test showed 96.4% agreement with the Panther Fusion SARS-CoV-2 Assay [56]. The Panther Fusion SARS-CoV-2 Assay showed 98.3% overall agreement with a laboratory-developed assay detecting the E gene of the virus [57]. The Roche Cobas assay showed very good agreement with the Cepheid assay on samples with high cycle thresholds [58].

It is worth noting, however, that a positive PCR result does not necessarily indicate the presence of infectious virus particles. A study suggested that a cycle threshold (Ct) equal to or more than 24 after 8 days from symptoms onset might predict the presence of noninfectious virus particles [59]. Another study failed to isolate virus particles in culture from samples containing 100,000 virus particles per milliliter of sputum after 10 days from symptom onset, and therefore suggested the discharge of a patient with these criteria [60]. These studies provide a guide for infection control and help in reducing the hospitalization period.

### 3.2 Droplet digital polymerase chain reaction (ddPCR)

Droplet digital PCR (ddPCR) is quantitative PCR utilizing standard PCR components with two main differences: first, the PCR reaction is performed in thousands of individually fractionated reaction droplets and second, the reaction results is read at an endpoint, unlike the real-time PCR [61]. These two features provide the ddPCR with multiple advantages over the real-time PCR such as: the direct and reproducible quantification of the target without the need for a standard curve, the potential for greater target multiplexing, and due to dilution, negation of the effect of PCR inhibitors, allowing the amplification of target with extremely low starting quantities [61].

In a comparison between real-time PCR and ddPCR for the detection of SARS-CoV-2, the ddPCR detected SARS-CoV-2 in 26 samples that were negative by real-time PCR and had improved sensitivity, negative predictive value, negative likelihood ratio, and accuracy [62]. Moreover, the ddPCR can also be used for the evaluation of disease progression through the monitoring of viral load in patient samples [63]. Two droplet digital PCR assays have been approved by the FDA for the detection of SARS-CoV-2 (Table 1 (Tab. 1)).

### 3.3 Loop-mediated isothermal amplification (LAMP) assays

The loop-mediated isothermal amplification (LAMP) assay was developed in 2000 to overcome the shortcomings of other nucleic acid amplification methods such as the need for complicated instruments for target amplification and detection of the product, as for PCR; the use of expensive modified nucleotides, as for strand displacement amplification; or compromised specificity due to low amplification temperature, as in self-sustained sequence replication and sequence-based amplification [64]. The LAMP assay utilizes 4–6 primers and a DNA polymerase with strand displacement activity. The primers work in sequential order during the LAMP cycles to produce an amplicon that makes a loop structure at its end due to the included complementary sequence in the design of the primers. This loop structure is used as a template with multiple amplification starting points which leads to the accumulation of large amounts of the target that can be detected by simple colorimetric methods [64]. These features make the LAMP assay rapid, sensitive, target-specific, and instrument-independent [65]. However, the primer design in LAMP is more complicated than in PCR, multiplexing of the target is limited, and the final product is not suitable for downstream amplification [65].

In combination with reverse transcription, the LAMP assay can also be used to detect RNA targets [64]. Numerous studies have described the design of LAMP assays for the detection of SARS-CoV-2, which have proven to be highly sensitive and have a similar specificity to that of RT-PCR [66], [67]. The limit of detection of the LAMP assay has been reported as approximately 100 RNA copies per reaction, and the turnaround time was less than 30 minutes [68], [69]. However, there are only two FDA-approved kits for diagnostic detection of SARS-CoV-2 based on LAMP technology. The AQ-TOP COVID-19 Rapid Detection Kit is a LAMP-based assay targeting the ORF1ab of SARS-CoV-2. The assay measures the fluorescence generated by fluorescence resonance energy transfer probes, which are integrated into the amplified products and, therefore, the results are given in real-time. The assay can be used on different thermocycler instruments and delivers positive results in 15 minutes, while it takes 30 minutes for a negative result. Despite the short time to result, this assay cannot be considered as rapid because it does not include RNA extraction, which must be performed separately.

The ID NOW COVID-19 assay from Abbott is another FDA-approved test based on LAMP technology (see Rapid Point-of-Care Assays below).

### 3.4 Rapid Point-of-Care Assays (POCT)

The need for prompt identification of SARS-CoV-2-infected individuals is crucial to put patients on appropriate management regimens and to reduce the transmission risk. Two rapid diagnostic assays were approved by the FDA for diagnostic use under emergency authorization (Table 1 (Tab. 1)). The ID NOW COVID-19 assay from Abbott employs the isothermal nucleic acid amplification technique directed towards a unique region in the RdRp region of the genome [70]. The test results can be delivered within 5–13 minutes according to the manufacturer’s instructions; therefore, the assay is very convenient as a point-of-care (POC) assay. The assay has 100% specificity and negative agreement with other rRT-PCR assays [71], [72], [73]. However, the assay detected 33%–45% less positive cases than the Xpress Xpert assay depending on the type of sample [70]. It also detected a lower number of positive samples when compared to the Abbott Real-Time SARS-CoV-2 assay and the Roche Cobas SARS-CoV-2 assay, especially in samples with a low viral load [54], [72]. Its overall sensitivity was estimated to be approximately 71.7% [73].

The Accula SARS-CoV-2 Test is another FDA-approved rapid test that can yield results within 30 minutes [74]. The assay employs RT-PCR techniques targeting the nucleocapsid gene of the virus; however, it reads the result via the lateral flow technique [74]. Similar to the Abbott ID NOW COVID-19 assay, the Accula SARS-CoV-2 showed 100% negative agreement when compared to assays targeting the E gene, while it had only 68.0% positive agreement especially in samples with a low viral load [74].

## 4 Immunological tests

As mentioned, the false-negative RT-PCR results for SARS-CoV-2, despite the presence of suggestive clinical disease manifestations, constitute a major challenge for the medical staff and increase the load on infection control officers and isolation rooms in hospitals. The humoral immune response against SARS-CoV-2 may help in confirming the diagnosis. Immunoglobulin M (IgM) and IgA are reported to appear within 5 days from symptom onset in 85.4% and 92.7% of patients, respectively [50], [51]. IgG antibodies are detected in 79% of the patients 14 days post-symptom onset [50], [51]. Combining both molecular and immunological tests was found to significantly increase the positive detection rate by more than 96% [50], [51], [75], [76].

Detection of SARS-CoV-2 antibodies has a number of advantages in addition to improving the total detection rate in COVID-19 patients. Antibody detection can help in estimating the actual disease prevalence in the population. Immunological assays detecting antibodies can be performed on a large scale, allowing for mass testing of the population. Furthermore, the detection of antibodies in COVID-19 patients may help in predicting disease prognosis. Patients with severe symptoms have been reported to have higher IgM antibody levels for a longer period of time [75], [77].

Immunological tests can detect either virus antigens or the antibodies against these antigens produced as an immune response to infection.

### 4.1 Antigen (Ag)-based immunoassays

This type of assay looks for virus proteins in respiratory samples from patients (such as nasopharyngeal swabs, oropharyngeal swabs, or nasal swabs) using an antibody specific to a certain target and different methods to detect the antigen-antibody (Ag-Ab) complexes, such as the immunofluorescence or lateral flow assays.

Multiple Ag-based immunoassays received the FDA approval under the emergency authorization such as the Sofia 2 SARS Antigen FIA, BinaxNOW COVID-19 Ag Card, and BD Veritor System for Rapid Detection of SARS-CoV-2 (Table 2 (Tab. 2); complete list: [78]). These assays are designed as POC tests providing results within 15 minutes. Many of these assays have not been validated by enough studies yet; however, they will have an important utility in the rapid diagnosis of patients admitted to the emergency department in hospitals. A recent comparison of the BD Veritor SARS-CoV-2 and the Sofia 2 SARS Antigen FIA tests, relative to the PCR results, found that these assays had 98.1% overall percent agreement with each other. The Veritor test showed 81.8% to 87.5% agreement with the PCR results, improving at increasing days from symptom onset [79]. Another study estimated the analytical sensitivity of the Abbott BinaxNOW COVID-19 Ag Card to be 40,000 to 80,000 copies/swab [80].

### 4.2 Antibody (Ab)-based immunoassays

A humoral immune response including IgM, IgG, and IgA antibodies can be measured by serological tests such as enzyme immunoassays (EIA). Numerous forms of EIA are available on the market. The FDA approved three types of such assays for testing of patients with COVID-19: the rapid immunoassays, Chemiluminescence immunoassays (CLIA), and the enzyme-linked immunosorbent assays (ELISA).

#### 4.2.1 Rapid immunoassays

Some rapid immunoassays employ lateral flow technology to detect the presence of IgM, IgG, or most commonly IgG and IgM antibodies simultaneously, against SARS-CoV-2. These assays are based on the liquid chromatography principle whereby the antibodies in patient samples migrate in flow with the buffering reagents on a nitrocellulose membrane. Detection components are immobilized on certain pre-marked locations on the nitrocellulose membrane. The rapid immunoassays can deliver results within 15 minutes and are simple, can be performed without the need for expensive equipment, and show good performance in comparison to conventional immunoassays [81]. The multiple advantages of rapid immunoassays encourage their use in the diagnosis of SARS-CoV-2 in clinical laboratories, especially in countries with limited resources [82]. Many commercially available rapid immunoassay kits received FDA approval for the detection of SARS-CoV-2 antibodies under emergency authorization (Table 2 (Tab. 2)). The rapid immunoassays are usually highly specific; however, they only have acceptable sensitivity [83], [84], [85], [86], [87].

#### 4.2.2 Enzyme immunoassays (EIA)

The most commonly used EIAs in clinical laboratories are the enzyme-linked immunosorbent assay (ELISA) and the Chemiluminescence immunoassay (CLIA). ELISA and CLIA are sensitive assays and can be used to test a large number of samples in one run, which makes them suitable for laboratories with high throughput requirements. They are also available in fully automated system formats and in manual formats. Some CLIAs have an advantage over ELISA in terms of turnaround time and analytical sensitivity. These assays can be designed to detect total SARS-CoV-2 antibodies or antibodies against one of its protein components such as the S protein or the nucleocapsid protein. It has been reported that the EIAs that detect anti-nucleocapsid protein antibodies provide better sensitivity than those that detect anti-spike protein antibodies [88]. However, since the nucleocapsid protein is one of the conserved proteins among coronaviruses, further studies are required to investigate whether this preferential sensitivity from anti-nucleocapsid protein antibodies is not due to cross-reactivity with other coronaviruses.

The enzyme immunoassays, in general, have shown very good performance in the detection of antibodies after day 10 of infection, with compromised sensitivity during days 5–9 of the infection [89], [90]. This is not due to a technical problem in the established assays but rather due to the immunological window required until antibodies are produced, which differs from patient to patient.

### 4.3 Virus neutralization assay (VNA)

While other immunoassays detect the total antibody spectrum against a virus or certain of its components, the virus neutralization assay (VNA) distinguishes the fraction of antibodies that interfere with the binding of the virus to its cellular receptor, inhibits cell entry, and neutralizes its infectivity; hence called neutralizing antibodies. In case of SARS-CoV-2, the neutralizing antibodies are directed against the receptor-binding domain (RBD) in the spike protein of the virus. The VNA is very helpful during the emergence of a new virus when no commercial assays are available to detect the presence of virus-specific antibodies. It is also helpful in determining whether a convalescent plasma contains protective neutralizing antibodies against the virus [91].

Conventional VNA is performed by mixing the whole virus in cell culture supernatant with the serum of a patient and measuring the virus infectivity to target cells compared to a control without patient serum. The test results are then measured by the level of damage the virus causes to the cell (cytopathic effect) [92]. However, this form of the assay should be performed in a biosafety level-3 (BLS-3) laboratory. To avoid handling infectious virus particles, a modified VNA based on a pseudovirus bearing the outer membrane of the infectious virus to be studied and all other components from other non-human pathogen virus or a mutated virus are used [93]. The readout in this case is a fluorescence such as green fluorescent protein or luciferase measurements in the infected cells. This modified assay can then be performed in a BSL-2 laboratory. The assay can also quantitatively determine the titer of antibodies by diluting the patient samples and reporting the lowest dilution of sample inhibiting virus or pseudovirus entry.

Despite the diagnostic and research utility of the VNA, it requires cell culture facility and specially trained personnel and therefore would not be convenient for commercial production.

## 5 Conclusions

Prompt diagnosis of COVID-19 cases is crucial for isolation, treatment, and contact tracing. Molecular diagnostic assays constitute by far the first line of defense for the identification of infected individuals. Immunological assays are mostly negative at the beginning of symptoms and cannot therefore play a significant role in the diagnosis of infected patients, but rather in defining the immunological status of a population at risk, such as health care workers, or the general population, particularly with the reports of frequent asymptomatic infection.

We additionally urgently need to produce more sensitive assays to reduce false-negative results and their deleterious effects on the spread of the virus in the human population.

## Notes

### Competing interests

The author declares that he has no competing interests.

## Figures and Tables

**Table 1:**
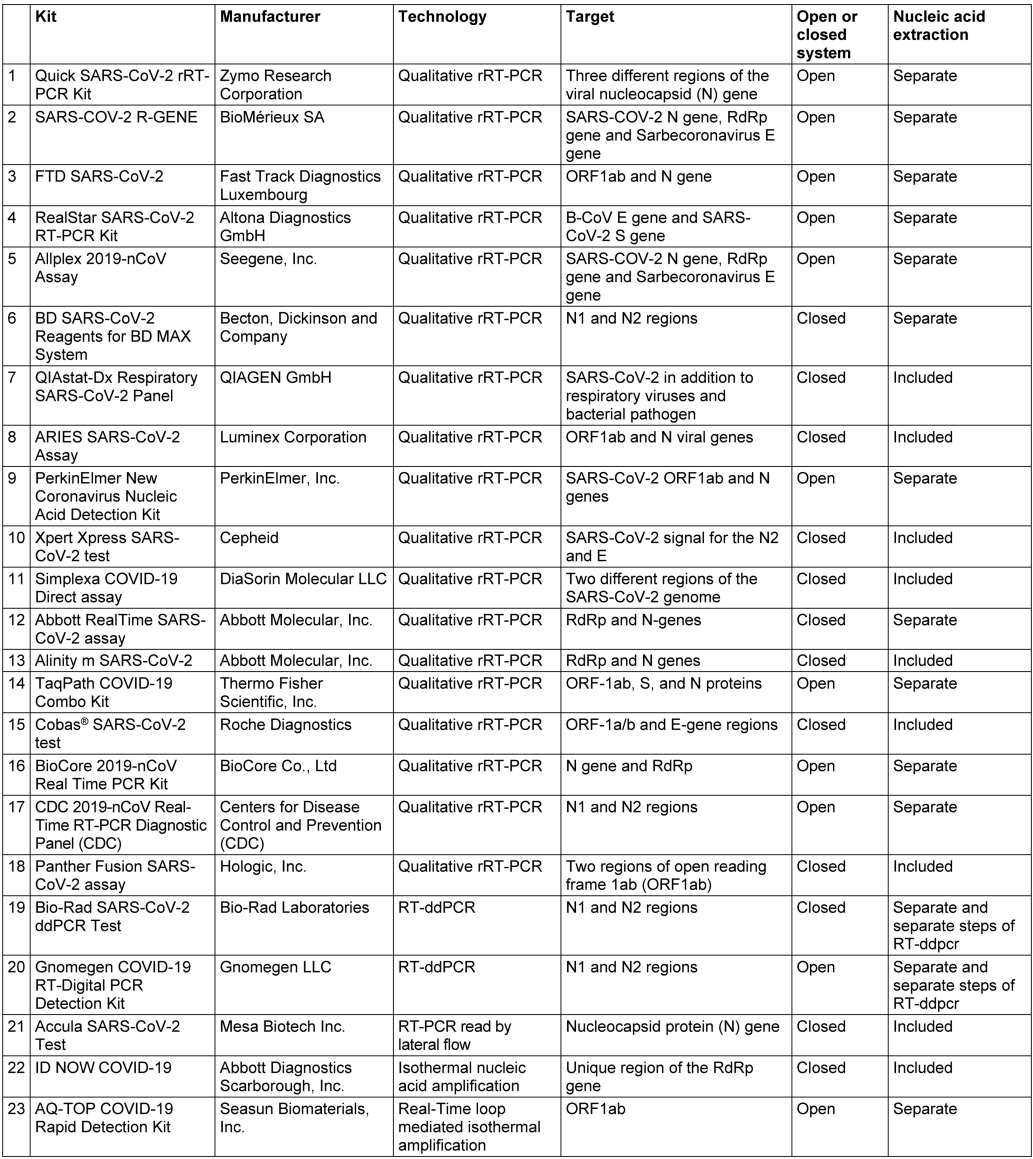
Examples of RT-PCR assays approved by FDA for COVID-19 diagnosis (full list: [93], last accessed 2020 Dec 21). The table describes a selected list of assays from the FDA-approved assay list based on their evaluation in the literature at the time of preparation of the manuscript or employment of different techniques bearing in mind the representation of different companies. Further information about the assays are found through the COVID-19 In Vitro Diagnostic Devices and Test Methods Database of the European Commission [94].

**Table 2:**
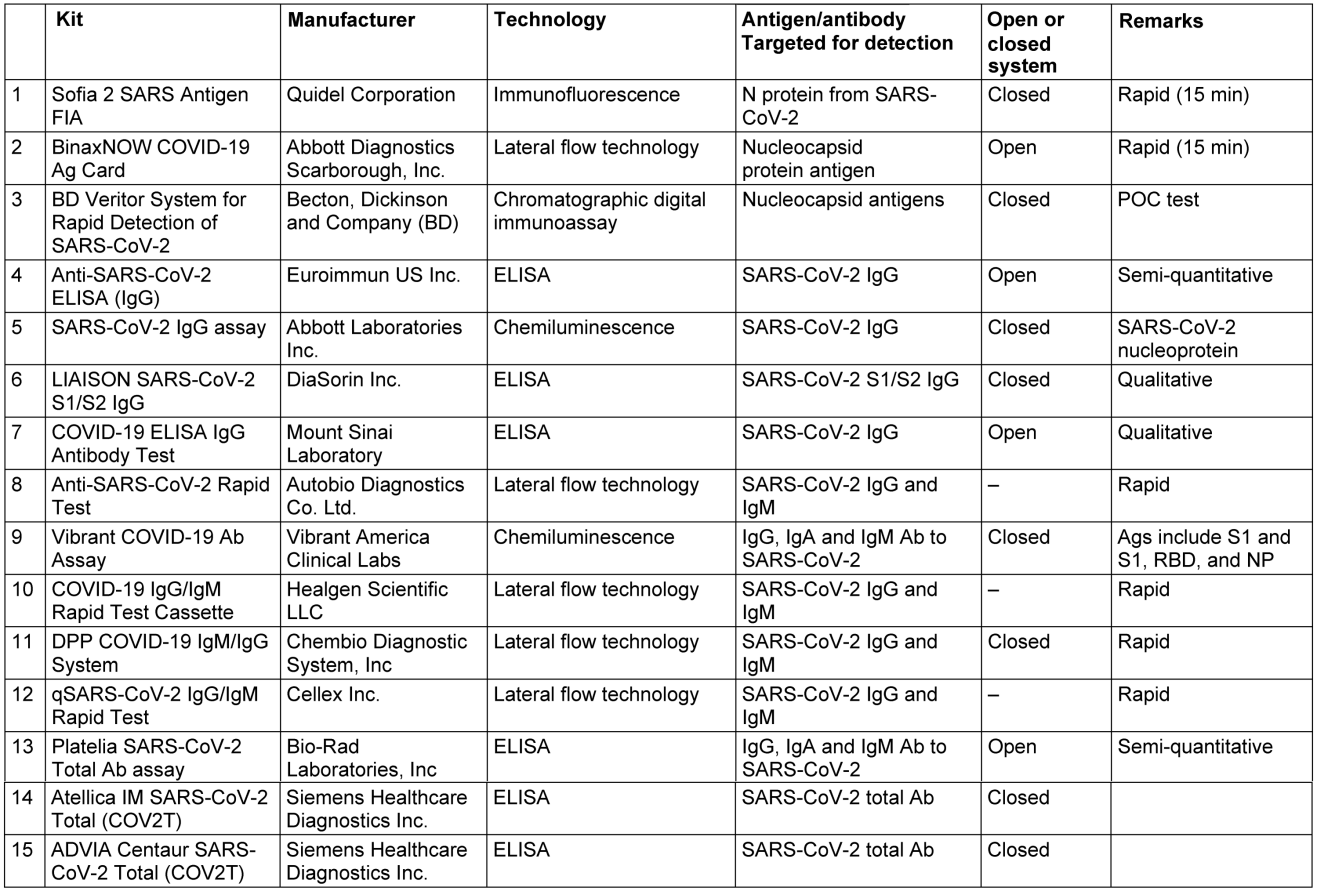
Examples of immunoassays approved by the FDA for the detection of SARS-CoV-2 antigens/antibodies (full list: [93], last accessed 2020 Dec 21). The table describes a selected list of assays from the FDA-approved assay list based on their evaluation in the literature at the time of preparation of the manuscript or employment of different techniques bearing in mind the representation of different companies. Further information about the assays are found through the COVID-19 In Vitro Diagnostic Devices and Test Methods Database of the European Commission [94].
